# Supplementary material for: BRAVE: a highly accurate method for predicting HIV-1 antibody resistance using large language models for proteins
Source: bioRxiv. 2025 Jul 31:2025.07.28.667234. Preprint. [Version 1] doi: 10.1101/2025.07.28.667234 (PMC12443041; doi:10.1101/2025.07.28.667234)
Supplement: 1 [file NIHPP2025.07.28.667234V1-supplement-1.pdf]

was not certified by peer review) is the author/funder. This article is a US Government work. It is not subject to copyright under 17 USC 1
and is also made available for use under a CC0 license.

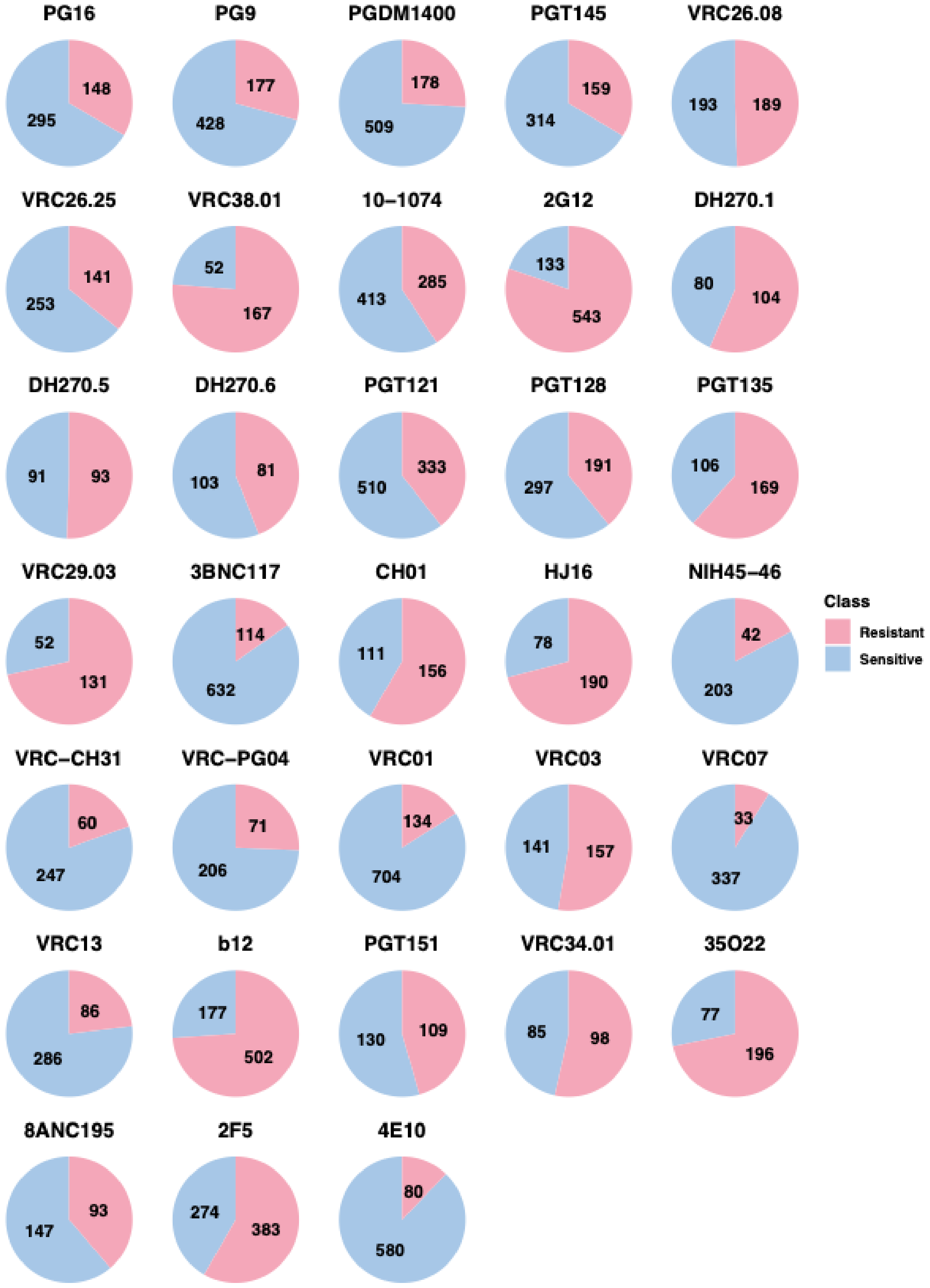

Figure S1. Training sets class distributions. Pie charts showing the distribution of "Resistant" and "Sensitive" classes for each bNAb training data, highlighting different imbalance ratios.

was not certified by peer review) is the author/funder. This article is a US Government work. It is not subject to copyright under 17 USC 1
and is also made available for use under a CC0 license.

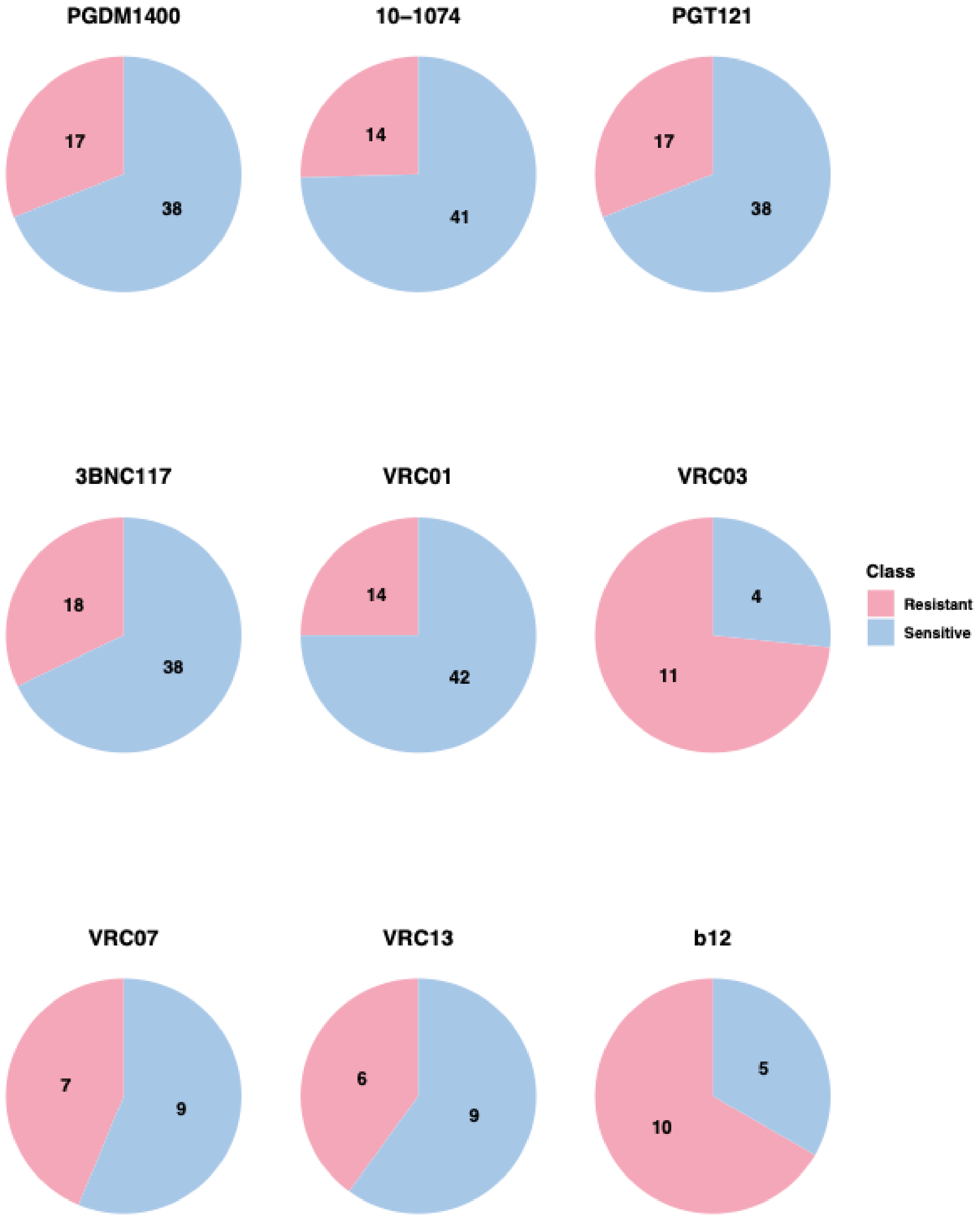

Figure S2. Test sets class distributions. Pie charts showing the distribution of "Resistant" and "Sensitive" classes for each bNAb test data, highlighting different imbalance ratios.
